# Vaccine Therapy

**Published:** 1910-11

**Authors:** 


**Vaccine Therapy.** Its Theory and Practice. By **R. W. Allen**, M.D., London. Third edition. Small octavo, pp. 287. Philadelphia: P. Blakiston's Sons & Co. 1910. (Cloth, $2.00.)

Another revision has given the author an opportunity to review all recent study and experimentation on the subject. He presents his conclusions in a completely rewritten book. Just what role is performed by "opsonins," tysins, stimulins, or by whatever name they may be called, in enabling the phagocytic cell to complete the destruction of the invading and infecting bacteria no one seems to be prepared to say; and opinions on the subject are far from unanimous. That such a substance does exist in the blood and materially assists in the destruction of bacteria seems to be beyond question. All that is known about it is fully set forth in this book. J. A. R.

